# A Split-and-Merge-Based Uterine Fibroid Ultrasound Image Segmentation Method in HIFU Therapy

**DOI:** 10.1371/journal.pone.0125738

**Published:** 2015-05-14

**Authors:** Menglong Xu, Dong Zhang, Yan Yang, Yu Liu, Zhiyong Yuan, Qianqing Qin

**Affiliations:** 1 School of Physics and Technology, Wuhan University, Wuhan, Hubei, China; 2 School of Computer, Wuhan University, Wuhan, Hubei, China; 3 State Key Laboratory of Information Engineering in Surveying, Mapping and Remote Sensing, Wuhan University, Wuhan, Hubei, China; Kermanshah University of Medical Sciences, IRAN, ISLAMIC REPUBLIC OF

## Abstract

High-intensity focused ultrasound (HIFU) therapy has been used to treat uterine fibroids widely and successfully. Uterine fibroid segmentation plays an important role in positioning the target region for HIFU therapy. Presently, it is completed by physicians manually, reducing the efficiency of therapy. Thus, computer-aided segmentation of uterine fibroids benefits the improvement of therapy efficiency. Recently, most computer-aided ultrasound segmentation methods have been based on the framework of contour evolution, such as snakes and level sets. These methods can achieve good performance, although they need an initial contour that influences segmentation results. It is difficult to obtain the initial contour automatically; thus, the initial contour is always obtained manually in many segmentation methods. A split-and-merge-based uterine fibroid segmentation method, which needs no initial contour to ensure less manual intervention, is proposed in this paper. The method first splits the image into many small homogeneous regions called superpixels. A new feature representation method based on texture histogram is employed to characterize each superpixel. Next, the superpixels are merged according to their similarities, which are measured by integrating their Quadratic-Chi texture histogram distances with their space adjacency. Multi-way Ncut is used as the merging criterion, and an adaptive scheme is incorporated to decrease manual intervention further. The method is implemented using Matlab on a personal computer (PC) platform with Intel Pentium Dual-Core CPU E5700. The method is validated on forty-two ultrasound images acquired from HIFU therapy. The average running time is 9.54 s. Statistical results showed that SI reaches a value as high as 87.58%, and normHD is 5.18% on average. It has been demonstrated that the proposed method is appropriate for segmentation of uterine fibroids in HIFU pre-treatment imaging and planning.

## Introduction

Uterine fibroids are one of the commonest benign tumors to occur among women, with an estimated incidence rate of 20–40% of women during their reproductive years [1]. Uterine fibroids can cause significant morbidity such as heavy menstrual bleeding and pelvic pressure [2]. Additionally, they seriously threaten women’s health and influence their quality of life. The traditional treatment for uterine fibroids is hysteromyomectomy, which can cause extensive pain in women both physically and mentally. Recently, high-intensity focused ultrasound (HIFU) with its noninvasive characteristic has been used to treat uterine fibroids widely and successfully [3–5].

Image guidance in HIFU therapy plays an important role because it offers the ability to monitor treatment accurately [6–7]. The two most popular methods of guidance are MRI based and ultrasound based [3]. Both methods have been used in HIFU therapy for uterine fibroids, and each has its advantages and disadvantages. Ultrasound guidance is generally applied because of its extensive availability, real-time visualization capabilities, flexibility and low cost [7–8]. In this paper, we focus on segmentations of uterine fibroids in ultrasound guidance images. In HIFU therapy, we have to maximize its action on the target and minimize its effect on the surrounding organs or tissues [9], indicating that accurate segmentation is needed. Although the patient always undergoes various preoperational examinations to obtain information about the tumor, the tumor region that needs to be ablated is positioned by real-time updated ultrasound images during ablation. At present, the localization of the target region is always achieved by physicians segmenting the tumor manually, a procedure that is onerous and decreases the efficiency of therapy. Thus, it is significant to propose a computer-aided uterine fibroid segmentation method in HIFU therapy that can relieve physicians’ burdens and improve therapy efficiency.

It is a challenging task to segment ultrasound images automatically because ultrasound images always have low quality. The ultrasound image quality suffers from the speckle effect, signal attenuation, acoustic shadows and a low signal-to-noise ratio (SNR). In the past decades, many methods have been proposed to segment ultrasound images [10], and many of them are within the framework of contour evolution. The basic idea of contour evolution is to minimize a given energy function beginning from an initial contour to obtain the final tumor contour. The contour evolution method can integrate multiple features to acquire satisfying results, such as intensity distribution [11–13], shape [13] and phase [14]. However, a poorly defined initial contour apparently results in inaccurate segmentation of ROIs (regions of interest) [15], and automatically generating a suitable initial contour is very difficult [16]. Another group of schemes to segment ultrasound images is to generate tumor contour from regions that are formed by clustering pixels. The scheme does not need an initial contour, which can reduce manual intervention. The popular way is to merge pixels pairwise according to their similarity until they cannot be merged anymore. The key issues are construction of the similarity measure and determining when to stop the procedure of merging. Wong and Scharcanski [17] proposed a prostate lesion segmentation method. The similarity measure is the likelihood function formed by regional statistics. The condition of stopping mergence is determined empirically. Huang et al [15] proposed a robust graph-based (RGB) ultrasound segmentation method for breast tumors; later, a parameter-optimization method was incorporated into this method [18]. The similarity measure and stop condition are formed according to the mean and standard deviation of the pixel intensities. Other ways of clustering are also applied in ultrasound image segmentation. Gao *et al* proposed a breast tumor segmentation method based on two-way normalized cut (Ncut) [19]. Hassan *et al* proposed an improved fuzzy c-means clustering method and applied it in a decision system for plaque detection in the carotid artery [20]. These methods can also integrate multiple features easily. One key issue for these methods is to determine a suitable number of clusters. In these methods, the number of clusters is determined manually and is always a fixed number. For images with complex structures, such as uterine fibroids, it would hardly be possible that a fixed number of clusters can be appropriate for all images.

**Fig 1:**
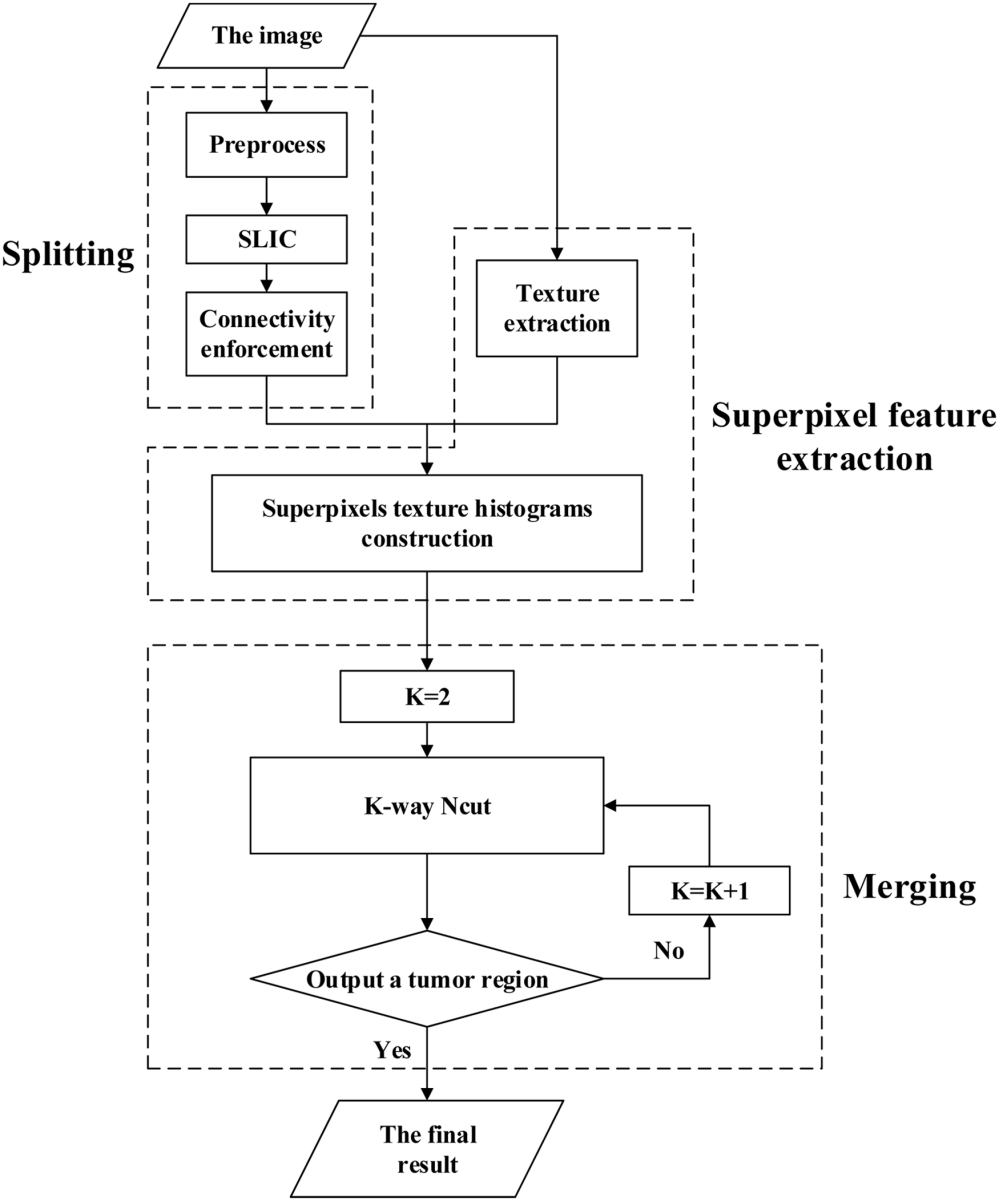
Flowchart of the proposed method.

In this paper, we intend to solve the problem of uterine fibroid segmentation in HIFU therapy, a procedure that has been reported scantily in the literatures. Considering that segmentation is applied in HIFU therapy, we adopted a region-based scheme to reduce manual intervention. We propose a split-and-merge segmentation method. Initially, the image is split into many small homogeneous subregions called superpixels. Next, those superpixels are merged according to the multi-way Ncut criterion. As the uterine fibroid always appears as heterogeneous in ultrasound images [21], it will be oversegmented into several superpixels. Here, we use a hybrid method based on SLIC (simple linear iterative clustering), which is efficient to obtain superpixels. Texture analysis has proven successful in ultrasound segmentation [10]. A new feature representation for superpixels is proposed based on texture histograms. We extract textures for each pixel by stacking gray values in a fixed window. Next, the texture histogram of each superpixel is constructed based on the extracted textures. Similarity between two superpixels is measured by the combination of their texture histogram Quadratic-Chi distance and space adjacent relationship. Ncut, as an effective criterion based on spectral clustering, is chosen at the procedure of merging. In image segmentation, Ncut always clusters pixels, whereas it clusters superpixels according to their similarities in our method. The introduction of superpixels reduces the computation complexity in clustering compared with clustering pixels directly. In addition, an adaptive strategy for selecting the number of clusters is integrated to Ncut to ensure a reasonable result of mergence. The adaptive scheme decreases manual intervention further and can output the tumor region automatically to obtain the final result. The entire process is summarized in Fig 1. To present the method clearly, the paper is organized as follows. First, we will provide details on the ultrasound images and proposed segmentation method. Next, we will display the segmentation results with exhaustive analysis. In the final section, a clear conclusion will be provided.

## Materials and Methods

### Materials

**Fig 2:**
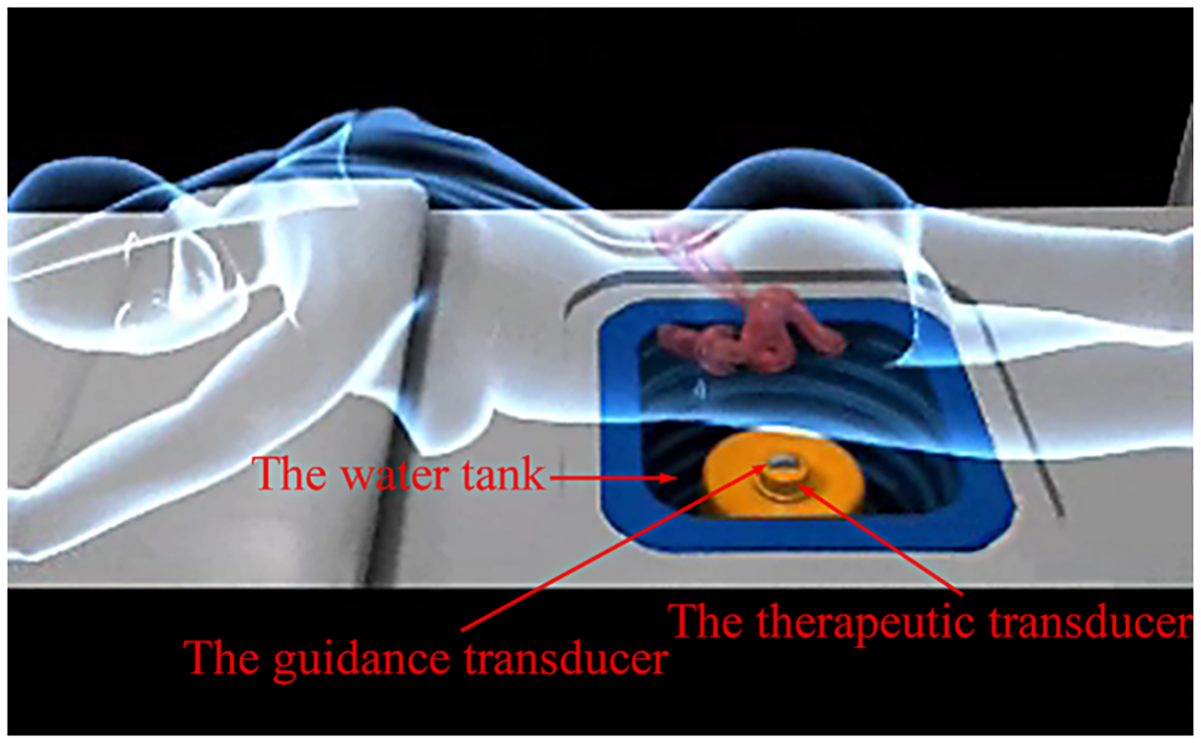
Illustration of the geometry and configuration of the transducers in the HIFU machine.

Forty-two ultrasound uterine fibroid images of different patients acquired from HIFU machines (Model JC200; Chongqing Haifu Tech Co., Ltd., Chongqing, China) at the First Affiliated Hospital of Chongqing Medical University (Chongqing, China) were used for experiments. Only the image data and corresponding tumor size information from the pre-operational examination were needed for our study; thus, personally identifiable information was deleted prior to the authors receiving the image data. Uterine fibroids comprise many types, including intramural fibroids, subserosal fibroids and submucosal fibroids; most fibroids are of mixed types [2]. Uterine fibroids appear as well-defined, heterogeneous and hypoechoic masses on ultrasound images frequently [21]. However, in some special cases, they may have complex sonographic appearance including iso- or hyper-echoic structures. For example, fibroids with calcification may have hyper-echo textures. According to the statistics of 1,114 HIFU-treated cases at the First Affiliated Hospital of Chongqing Medical University, 68% of the uterine fibroids were intramural fibroids, 23% were subserosal fibroids, and 9% were submucosal fibroids. Most of the cases were hypo-echoic, and the hyper-echoic cases were seldom encountered. The purpose of our study is to segment tumor regions from the HIFU guidance images using a computerized algorithm instead of manual delineation, rather than help the radiologist detect the lost tumor boundaries, in which *a priori* knowledge is generally needed. To ensure safety, it is better to position the tumor region in the HIFU ablation process with less information other than the guiding image itself. Thus, the test images were selected by a radiologist based on the criterion that they are typical ultrasound guidance images in HIFU therapy and can be used to position the tumors for HIFU ablation directly. Specifically, tumors in thirty-five test images had a relatively clear boundary, indicating that the tumor boundary can be recognized primarily dependent on the ultrasonic appearance but not highly dependent on *a priori* knowledge, such as information concerning biological structure and blood supply. Another seven images, in which the tumors had weak boundaries were chosen to test the adaptability of the proposed method. Among forty-two images, only one tumor had a hyper-echoic appearance; the remainder had a hypo-echoic appearance. The JC200 HIFU machine is integrated with B-mode ultrasound equipment (Esaote, Italy) for guiding purposes. A diagnostic transducer (CA430; Esaote, Italy; convex array, 192 elements, 3.5-MHz center frequency, 1-mm nominal axial spatial resolution, 2-mm lateral spatial resolution) for guidance imaging is installed in front of a therapeutic transducer in the machine. The combination of transducers is placed in a sink filled with water because the transducers cannot contact the patient’s skin directly during the therapeutic process so that water is used to eliminate the obstruction of air to ultrasound waves. Fig 2 shows a typical configuration of combined guidance and therapy transducers in the water tank used in this study. The mounting mode of the guidance transducer influences the image quality. It cannot be improved by the clinician adjusting the probe, such as compressing the skin and adjusting the incident angle. Moreover, the reflection from the interface between the skin and water always degrades the image quality.

**Fig 3:**
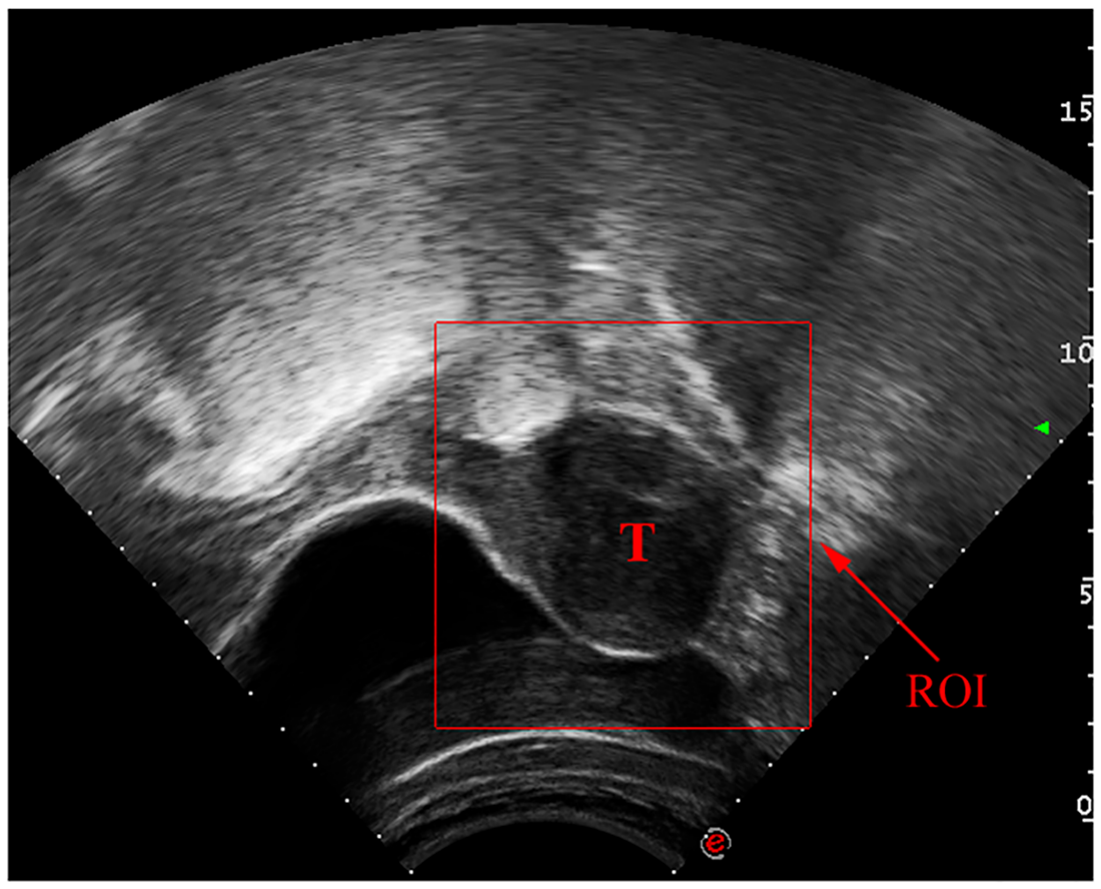
Illustration of the original image with an ROI. T: Tumor.

The original image size from HIFU machines is 768×576 (pixels). Radiologists set an ROI for each image, and these ROI images are segmented automatically by the proposed method. The ROI is set as a rectangular region that can cover the tumor entirely and make the tumor be located centrally. Because the ultrasound scanning area is displayed as a sector (Fig 3) other than a regular rectangle in the original image, it is better to draw the ROI as small as possible to exclude regions outside the scanning sector. As the tumor size varies, the ROI image size varies. The average ROI image size is 223×255 (pixels). Fig 3 provides an illustration of the ROI drawn on the original image. The uterine fibroid is heterogeneous, and its boundary is blurred. Additionally, in many images, not only tumor and uterine tissues around the uterine fibroid but also other organs appear, even in ROI images. The complex structure of the image makes it difficult to determine a suitable number of clusters.

### Splitting method

#### Preprocessing method

The procedure of splitting generates superpixels based on the gray level and position information. The ultrasound image is always filled with noise, which influences the performance of the splitting algorithm. Thus, an appropriate preprocess is needed. Bilateral filtering [22] as a nonlinear filter has been widely used in digital image processing. We apply it in ultrasound denoising because it can preserve edges while denoising. The idea of bilateral filtering is to consider both the space domain and value domain effects when smoothing. Given an input image *I*(*x*,*y*), where (*x*,*y*) is the space coordinate, the filtered image can be described as follows:

$$I^{f}(x,y)=\frac{\sum_{i,j}I(i,j)w^{f}(x,y,i,j)}{\sum_{i,j}w^{f}(x,y,i,j)}$$ (1)

$$w^{f}(x,y,i,j)=\exp(-\frac{{\left(x-i\right)}^{2}+{\left(y-j\right)}^{2}}{2\sigma_{d}^{2}})\exp(-\frac{{\left\|I(x,y)-I(i,j)\right\|}^{2}}{2\sigma_{r}^{2}})$$ (2)

where (*i*,*j*) represents pixels in a window with the size of *S* ^*f*^×*S* ^*f*^ around (*x*,*y*), and *σ*_*d*_ and *σ*_*r*_ are parameters of weight function *w* ^*f*^. For homogeneous regions, the filter behaves like the Gaussian low-pass filter. When pixels are at the edges, the introduction of the value kernel makes the edges preserved.

#### Superpixel generation

Superpixels are a group of pixels with a homogeneous appearance and are a more perceptually meaningful form of image representation. The use of superpixels can reduce the complexity of subsequent image processing. Superpixels have been widely used in many aspects of computer vision. Many superpixel algorithms have been proposed, including graph-based and gradient-ascent-based methods [23–25]. Recently, a new superpixel algorithm called SLIC was proposed, and its performances were compared with 5 state-of-the-art superpixel methods by the authors of SLIC [25] based on several widely used image databases, including the Berkeley database and Microsoft Research Cambridge database. The experimental results show that SLIC ranks at the top by the computing time and among the top two by the boundary adherence. Although the evaluation databases are mainly composed of natural images instead of ultrasound images, the segmentation speed will not differ obviously if SLIC is applied to ultrasound images. Because of the good boundary adherence of the SLIC algorithm on natural images, in which the object and background frequently manifest different texture appearances just as they do in ultrasound images, it is reasonable to expect that SLIC has a good boundary adherence in ultrasound images. Another study reported in [26] also showed that the computing speed and boundary performance of SLIC surpass that of two other superpixel algorithms according to the experiment results based on medical images, including ultrasound images. For the fastest segmentation speed and reasonable adherence, SLIC was adopted as the superpixel generation algorithm here.

For a gray-scale image *I* with the size of *N* pixels, we want to generate *M* superpixels {Ω_1_,Ω_2_,…,Ω_*M*_}, Ω_*p*_ ⋂ Ω_*q*_ = ∅ *p*,*q* ϵ [1,*M*] and Ω_1_ ⋃ Ω_2_ ⋃…⋃ Ω_*M*_ = *I*. The SLIC algorithm clusters pixels in a 3D space ℝ^3^: (*x*,*y*,*I*(*x*,*y*)), where *x*, *y* are space coordinates, and *I*(*x*,*y*) is the image gray value (intensity). Supposing that superpixels are approximately equal-sized squares, the area of each superpixel is approximately *N/M*, and the distance between superpixel centers is approximately $S=\sqrt{N/M}$. Initially, *M* pixels are selected evenly as initial cluster centers. Each pixel is assigned to its nearest cluster center, and *M* initial clusters are formed. Similar to the K-Means algorithm [27], the cluster centers and clusters are updated iteratively until they are converged. The new cluster center is obtained by calculating the average space coordinates and gray value, respectively, for pixels in a cluster. Once new cluster centers are obtained, each pixel can be reassigned to the nearest new cluster center in the next iteration so that new clusters are formed. However, unlike K-Means, the SLIC algorithm searching space is confined to a 2*S*×2*S* region around the cluster center, not the entire image, thus reducing computations. The distance measure *d* of two pixels *a* and *b* is as follows:

$$d_{g}=\sqrt{{\left(I(x_{a},y_{a})-I(x_{b},y_{b})\right)}^{2}}$$ (3)

$$d_{l}=\sqrt{{\left(x_{a}-x_{b}\right)}^{2}+{\left(y_{a}-y_{b}\right)}^{2}}$$ (4)

$$d=\sqrt{{\left(\frac{d_{g}}{cmp}\right)}^{2}+{\left(\frac{d_{l}}{S}\right)}^{2}}\;,$$ (5)

where (*x*_*a*_,*y*_*a*_) and (*x*_*b*_,*y*_*b*_) are coordinates of pixels *a* and *b*, and *cmp* is introduced as the compactness factor.

Because SLIC does not enforce connectivity explicitly, some clusters are not connective spatially. In the non-connective cluster, several orphaned pixels that are not connective to the main part of the cluster remains. To correct for it, these pixels are reassigned to its adjacent connective cluster with the most similar average gray value. After the correction, the number of superpixels and size of superpixels will be changed. This is deemed acceptable because the goal of splitting is to oversegment the image sufficiently, not to produce an accurate amount of small subregions.

**Fig 4:**
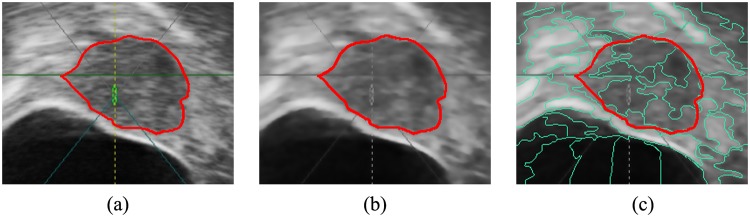
Splitting results. Red lines represent the tumor boundary depicted by the radiologist. (a) The original image. (b) The image after preprocess. (c) Superpixels.

Fig 4 shows the result of splitting. It can be seen that the tumor has been segmented into several superpixels. Superpixels boundaries fit well with the tumor boundary. Therefore, the following key issue to segment the tumor is to merge those superpixels belonging to the tumor. Our solution is to use multi-way Ncut to cluster superpixels by measuring their texture features.

### Superpixel feature extraction

**Fig 5:**
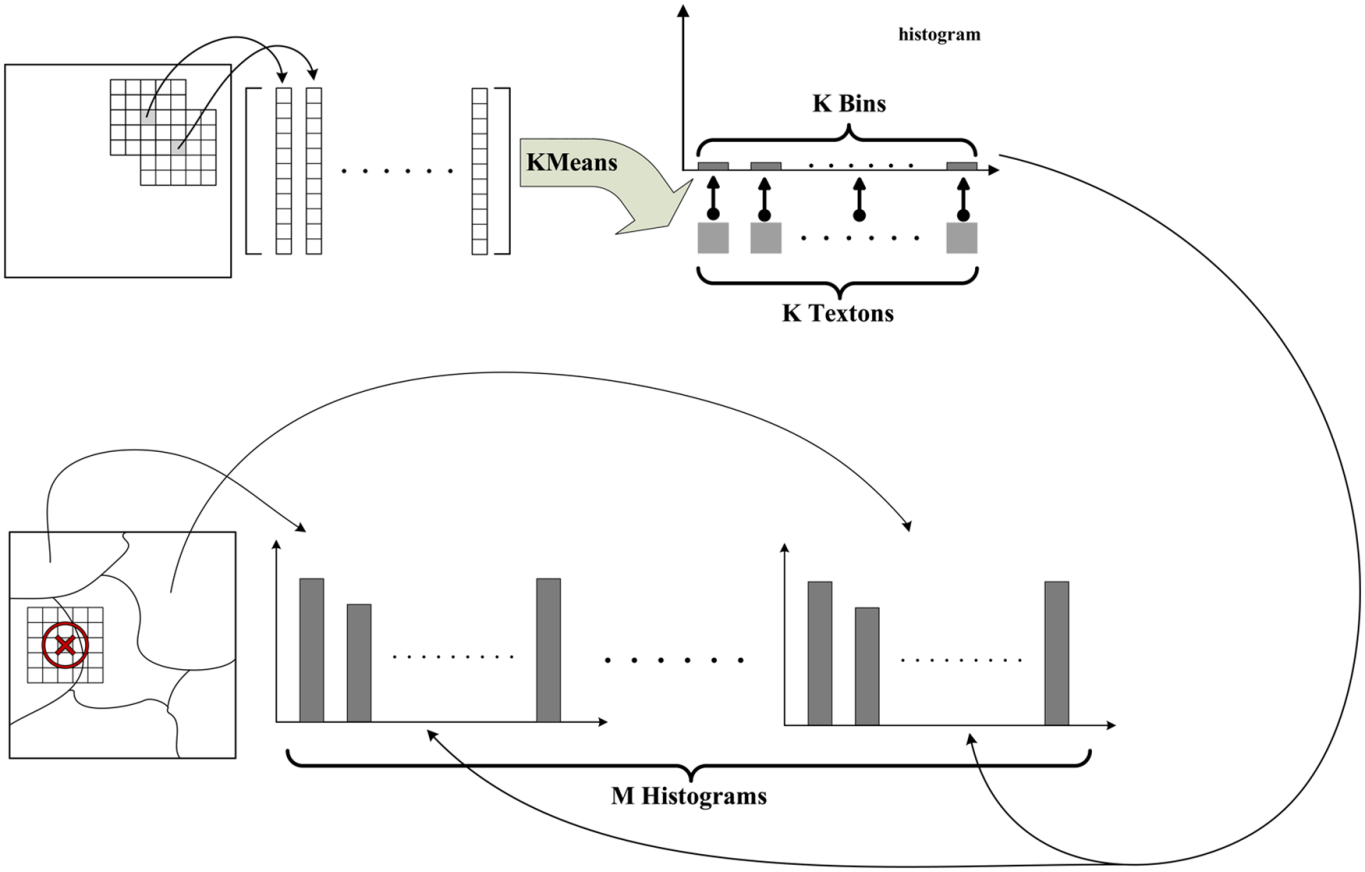
Diagram of superpixel feature extraction.

Texture is an important feature in image analysis and has been widely used in ultrasound image analysis. Texture analysis is always completed based on regional statistics; thus, it is suitable for extracting superpixel texture information. It is known that speckles can be regarded as an important factor to cause texture appearance in ultrasound images [10]. To preserve more information, the original image without filtering is used for extracting texture features. The superpixel feature extraction includes two steps: texture extraction for each pixel in an image, and texture histogram construction for each superpixel. The entire process is illustrated in Fig 5.

The texture of each pixel is extracted firstly. For a pixel *p*, we stack gray values inside a (2*r*+1)×(2*r*+1) window around the pixel and store them into a vector *T*(*p*) with the dimension of (2*r*+1)^2^. Using this method, we can obtain a texture model for each pixel in an image by its corresponding vector. Varma and Zisserman reported that this method of texture extraction is effective in texture classification and noted that it actually represents the joint PDF (probability distribution function) of the central pixel and their neighborhoods [28]. This texture representation has been used in natural image segmentation and achieves good performance [29–30]. From these literature sources, to employ texture features for image segmentation, it is supposed that the distribution of texture features is subject to a mixed Gaussian distribution that can be degenerated and of different dimensions. However, the intensity distribution of ultrasound images is complex [31] [32], and the distribution of their texture features cannot always be modeled as a mixed Gaussian distribution simply. To overcome this problem, we constructed a texture histogram for each superpixel to characterize its feature.

**Fig 6:**
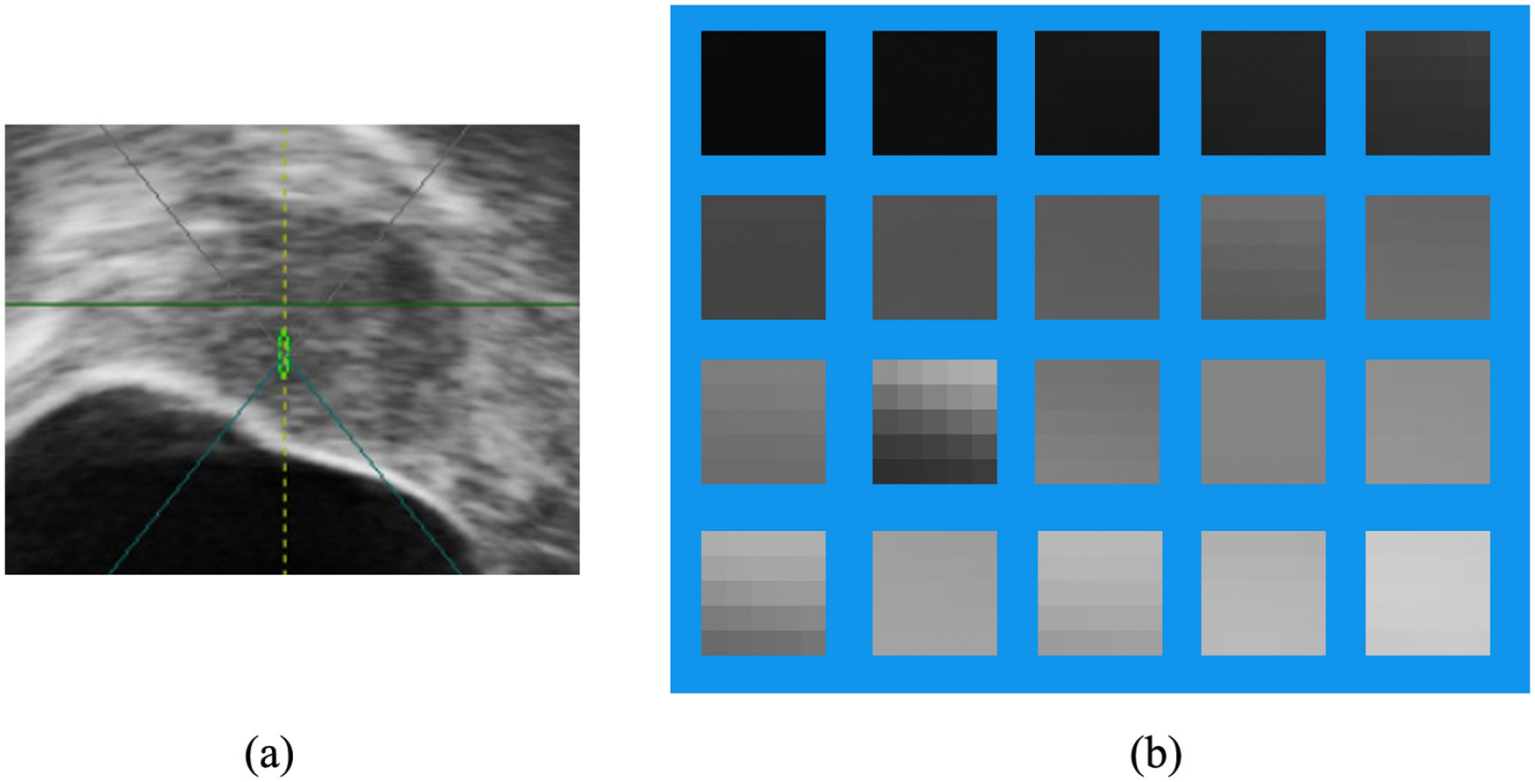
Examples of textons. (a) The image. (b) 20 corresponding textons.

The construction of histograms can be completed by two steps, defining bins (horizontal axis of the histogram) and determining the values of each bin (vertical axis). First, all texture vectors are classified into *K* clusters using the K-Means algorithm. Thus, a pixel *p* is assigned to a unique label *L*(*p*), indicating to which group the pixel texture belongs. Texture vectors belonging to the same cluster have similar characteristics, which can be characterized by the cluster center. The cluster center, whose representing vector is denoted as *T*_*c*_(*η*), can be called a texton [33]. Fig 6 shows an example of textons. The *K* bins of the histogram are formed naturally based on the *K* clusters. Next, the texture histogram for each superpixel is constructed by counting appearance times of each bin as follows:

$$hist_{m}(\eta )=\sum_{p\in \Omega_{m}}\delta \left(L(p),\eta \right)\;m\in [1,M],\;\eta \in [1,K]$$ (6)

$$\delta (L(p),\eta )=\{\begin{array}{cc} 1\;, & L(p)=\eta \\ 0\;, & L(p)\neq \eta \end{array}\;.$$ (7)

It should be noted that pixels whose windows span different superpixels are not counted as suggested in [29]. The histogram is normalized to [0,1]. The texture histogram has been widely used in texture analysis [19, 28, 33]. In these studies, the texture histogram was used in a pixel neighbor to characterize pixel texture information. It is natural to generalize texture histograms to describe superpixel texture information because superpixels are homogeneous. Because the superpixel includes many pixels, the texture histogram has much more statistical significance.

### Superpixel merging

#### The framework of the Ncut clustering method

Ncut [34] is a data-clustering method based on graph theory. Given a weighted undirected graph *G* = (*V*,*E*,*W*), where *V* is the set of $\tilde{V}$ vertices, *E* is the set of edges connecting vertices, and *W* is the weight matrix whose element *w*(*m*,*n*) represents the similarity between two vertices *m* and *n* in *V*. Supposing the vertices set *V* is partitioned into *F* sets {Φ_1_,Φ_2_,…,Φ_*F*_}, Φ_*c*_⋂Φ_*d*_ = ∅*c*,*d* ϵ [1,*F*], Φ_1_⋃Φ_2_⋃…⋃Φ_*F*_ = *V*. The best partition is to minimize

$$Ncut=\sum_{k=1}^{F}\frac{cut(\Phi_{k},V-\Phi_{k})}{assoc(\Phi_{k},V)}$$ (8)

$$cut(\Phi_{c},\Phi_{d})=\sum_{m\in \Phi_{c},n\in \Phi_{d}}w(m,n)\;c,d\in [1,F]$$ (9)

$$assoc(\Phi_{c},V)=\sum_{m\in \Phi_{c},n\in V}w(m,n)\;c\in [1,F]\;.$$ (10)

When *F* = 2, this is the classic two-way Ncut problem that can be solved by transforming it to a generalized eigenvalue equation (*D*-*W*)*x* = *λDx*, where *D* is a diagonal matrix with its diagonal elements $d(m)=\sum_{n}w(m,n)$. For *F*≥3, it can be described as multiclass spectral clustering, and some approaches have been proposed to solve it [33–36]. In general, *F-*generalized eigenvalues and their corresponding eigenvectors form an equation, which can be described generally as *Wx* = *λDx*, are needed to complete the partition. Here we use the so-called robust method—namely, it is robust to random initialization compared with other spectral clustering methods [35].

As $\frac{1}{F}(\sum_{k=1}^{F}\frac{cut(\Phi_{k},V-\Phi_{k})}{assoc(\Phi_{k},V)}+\sum_{k=1}^{F}\frac{cut(\Phi_{k},\Phi_{k})}{assoc(\Phi_{k},V)})=1$, minimizing *Ncut* is equal to maximizing

$$Nassoc=\frac{1}{F}\sum_{k=1}^{F}\frac{cut(\Phi_{k},\Phi_{k})}{assoc(\Phi_{k},V)}\;.$$ (11)

It can be solved by two steps. First, the problem is transformed into an eigenvalue problem to obtain a set of continuous global optima. Next, the closest discrete solution from the continuous optima is sought. The output is represented by a $\tilde{V}\times F$ binary matrix *X* whose element *X*(*v*,*k*) represents whether the node *v* belongs to the set Φ_*k*_. If *X*(*v*,*k*) = 1, the node *v* belongs to the set Φ_*k*_ and *X*(*v*,*k*) = 0 by contrast. The specific algorithm is listed below:

1. Obtain *F* largest eigenvalues and its corresponding eigenvectors {*x*_1_,*x*_2_,…,*x*_*F*_} by solving the equation $D^{-\frac{1}{2}}WD^{-\frac{1}{2}}x=\lambda x$, where *λ* denotes the eigenvalue, and *x* is its corresponding eigenvector.
2. Form a matrix *Z* by arranging column vectors as [*x*_1_,*x*_2_,…,*x*_*F*_].
3. Obtain $\hat{Z}=D^{-\frac{1}{2}}Z$, and normalize $\hat{Z}$.
4. Initialize an *F* × *F* matrix $\hat{R}$ by choosing *F* rows of $\hat{Z}$, which are as orthogonal to each other as possible.
5. Find the optimal discrete solution *X*.$\hat{X}=\hat{Z}\hat{R}$. *X* is obtained by NMS (non-maximum suppression) from $\hat{X}$ in the row direction.
6. Update the matrix $\hat{R}$ by $X^{T}\hat{Z}=U\Omega {\tilde{U}}^{T}$ and $\hat{R}=\tilde{U}U^{T}$. If $\bar{\phi }=trace(\Omega )$ is converged, stop and output the final solution *X*, otherwise go to step 5.

The Ncut algorithm has been widely applied in image segmentation for both natural [33–34] and ultrasound [19, 37] images. In these studies, the vertex in *V* represents the pixel in the image. In our application, the vertex in *V* represents the superpixel. The key problems of applying Ncut in our method are the construction of *W* and the choice of *F*.

#### The construction of matrix *W*

The elements of matrix *W* represent the similarity between two vertices. In the proposed method, the vertex in the graph represents the superpixel. Thus, the matrix *W* measures the similarity between superpixels. As each superpixel is characterized by a texture histogram, the basic form of matrix *W* element is

$$w(m,n)\;=\exp(-\frac{1}{\sigma }\frac{dis(hist_{m},hist_{n})}{\underset{m,n}{\max}(dis(hist_{m},hist_{n}))})\;,$$ (12)

where σ is a scale factor. The *dis*(*hist*_*m*_,*hist*_*n*_) denotes the distance between two histograms of superpixels Ω_*m*_ and Ω_*n*_. The normalized form $\frac{dis(hist_{m},hist_{n})}{\max(dis)}$ is used to confine it into [0, 1].

The *χ* ^2^ distance is always used to measure the difference between histograms [19, 28, 33]. For two histograms *hist*_*m*_ and *hist*_*n*_, their *χ* ^2^ distance is defined as

$$d_{\chi^{2}}=\sum_{\alpha }\frac{{\left(hist_{m}(\alpha )-hist_{n}(\alpha )\right)}^{2}}{hist_{m}(\alpha )+hist_{n}(\alpha )}\;,$$ (13)

where *hist*_*m*_(*α*)denotes the value of the *α*th bin of the histogram *hist*_*m*_. However, the *χ* ^2^ distance only considers the bin-to-bin distances, and it is better to consider cross-bin distances. The histogram can be regarded as a discrete form of the PDF. The performance of the bin-to-bin distances depends on the appropriately defined bins. Too many bins make the bin-to-bin distance not robust because a small amount of data changes will enlarge the difference of the histogram. In the proposed method, the histogram is constructed using K-Means. The cluster number of K-Means that determines the bin number in the histogram is always pre-defined as a parameter; thus, it may not be appropriate for each image. To avoid information loss, it is always chosen to be higher to circumvent histogram degeneracy. Thus, the distance that considers the cross-bin relationship should be adopted to improve the performance of the histogram distance. Here a cross-bin distance with less computation time called the Quadratic-Chi histogram distance was adopted [38]. The Quadratic-Chi histogram distance can be regarded as the combination of the Quadratic-Form distance and *χ* ^2^ distance. For two histograms *hist*_*m*_ and *hist*_*n*_, the distance between them is defined as

$$QC_{\xi }^{A}(hist_{m},hist_{n})=\underset{\alpha ,\beta }{\sum }(\frac{\left(hist_{m}(\alpha )-hist_{n}(\alpha )\right)}{{\left(\underset{\gamma }{\sum }(hist_{m}(\gamma )+hist_{n}(\gamma ))A_{\gamma \alpha }\right)}^{\xi }})(\frac{\left(hist_{m}(\beta )-hist_{n}(\beta )\right)}{{\left(\underset{\gamma }{\sum }(hist_{m}(\gamma )+hist_{n}(\gamma ))A_{\gamma \beta }\right)}^{\xi }})A(\alpha ,\beta )\;,$$ (14)

where *hist*_*m*_(*α*) denotes the value of the *α*th bin of the histogram *hist*_*m*_. *ξ* is the normalization factor and is set as 0.5 here to maintain consistency with the *χ* ^2^ distance. Actually, the *χ* ^2^ distance can be regarded as a special form of the Quadratic-Chi histogram distance because it can be obtained by $QC_{0.5}^{Id}/2$ directly, where *Id* represents the identity matrix. The matrix *A* in (14) is a non-negative symmetric matrix to measure the similarity of bins, indicating that its element *A*(*α*,*β*) denotes the similarity between the *α*th bin and *β*th bin. Because bins are formed from textons, the similarity of bins is the similarity of the corresponding textons of their cluster centers. Because *T*_*c*_(*η*) denotes the corresponding center vector of the *η*th bin, the matrix *A* can be defined as

$$A(\alpha ,\beta )=1-\frac{{\left\|T_{c}(\alpha )-T_{c}(\beta )\right\|}_{2}}{\underset{\alpha ,\beta }{\max}({\left\|T_{c}(\alpha )-T_{c}(\beta )\right\|}_{2})}\;.$$ (15)

Another issue that should be considered is the connectivity. The tumor is connective and cannot be interrupted by backgrounds. When using Ncut segmenting pixels directly, it is always ensured by restricting the similarity measurement to a certain geometric distance range [19, 33, 34, 37]. We use Ncut to cluster superpixels, and the connectivity can be enforced using the region adjacent matrix (RAM). We define an *M*×*M* binary RAM *B* for *M* superpixels. Its element *B*(*m*,*n*) denotes whether superpixel *m* is adjacent to superpixel *n*,

$$B(m,n)=\{\begin{array}{ll} 1\;, & \text{if superpixel }m\text{ is adjacent to }n\\ 0\;, & \text{otherwise} \end{array}\;.$$ (16)

**Fig 7:**
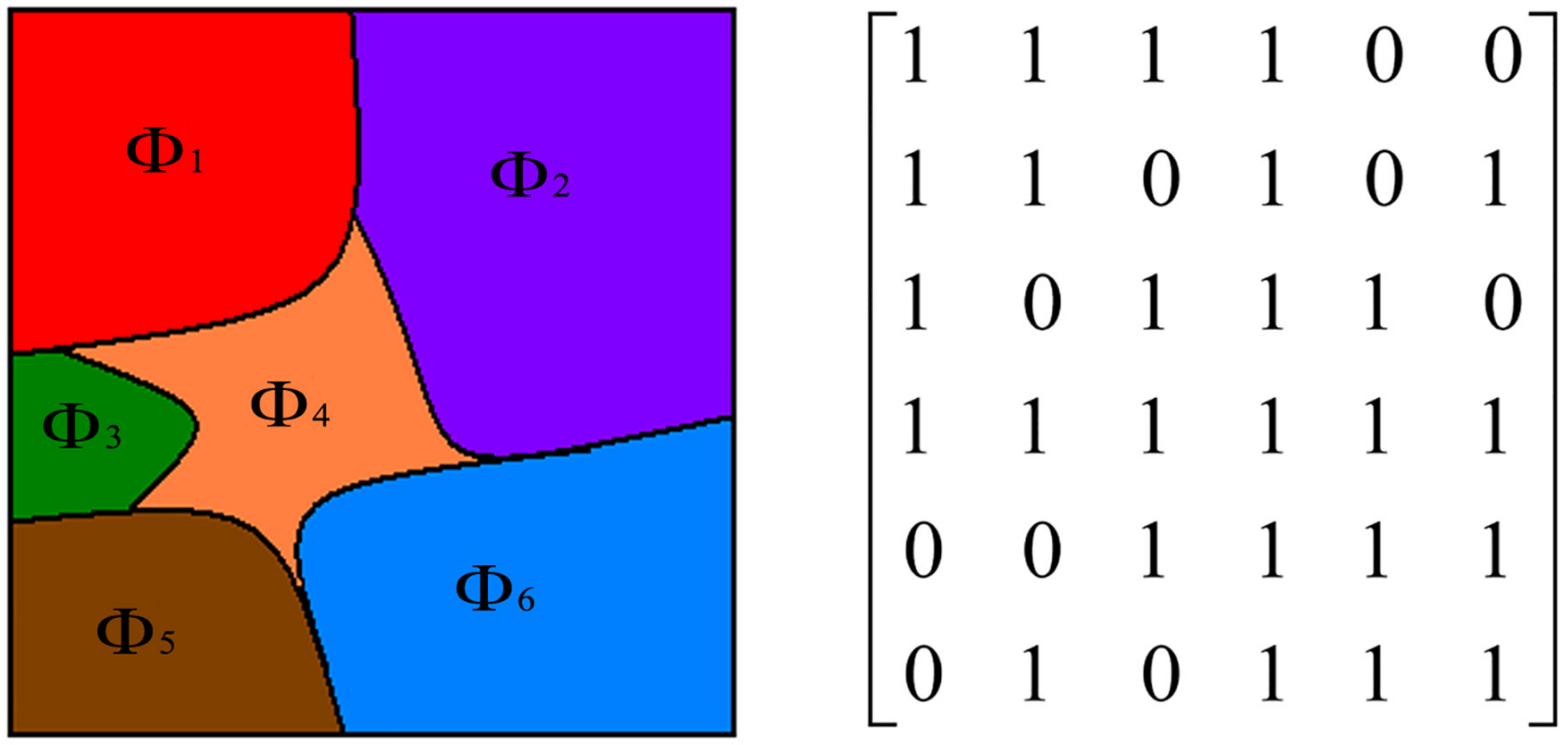
An example of RAM. Left: The image. Right: corresponding RAM.

Fig 7 shows an example. In [17, 29, 30], a similar region adjacency graph (RAG) scheme is used in the process of merging. Compared with RAG, our matrix form is easier to be integrated in the framework of Ncut. Moreover, the introduction of RAM can ensure the sparsity of the similarity matrix *W*; thus, the fast algorithm of an eigensolver such as the Lanczos method [39], which is an iterative solution to find the eigenvalues and eigenvectors computationally efficiently, can be used. Finally, by putting the above items together, the element of the weight matrix *W* is defined as

$$w(m,n)=\exp\left\{-\frac{1}{\sigma }\frac{QC_{0.5}^{A}(hist_{m},hist_{n})}{\underset{m,n}{\max}[QC_{0.5}^{A}(hist_{m},hist_{n})]}\right\}B(m,n)\;.$$ (17)

#### The adaptive choice of the number of clusters

**Fig 8:**
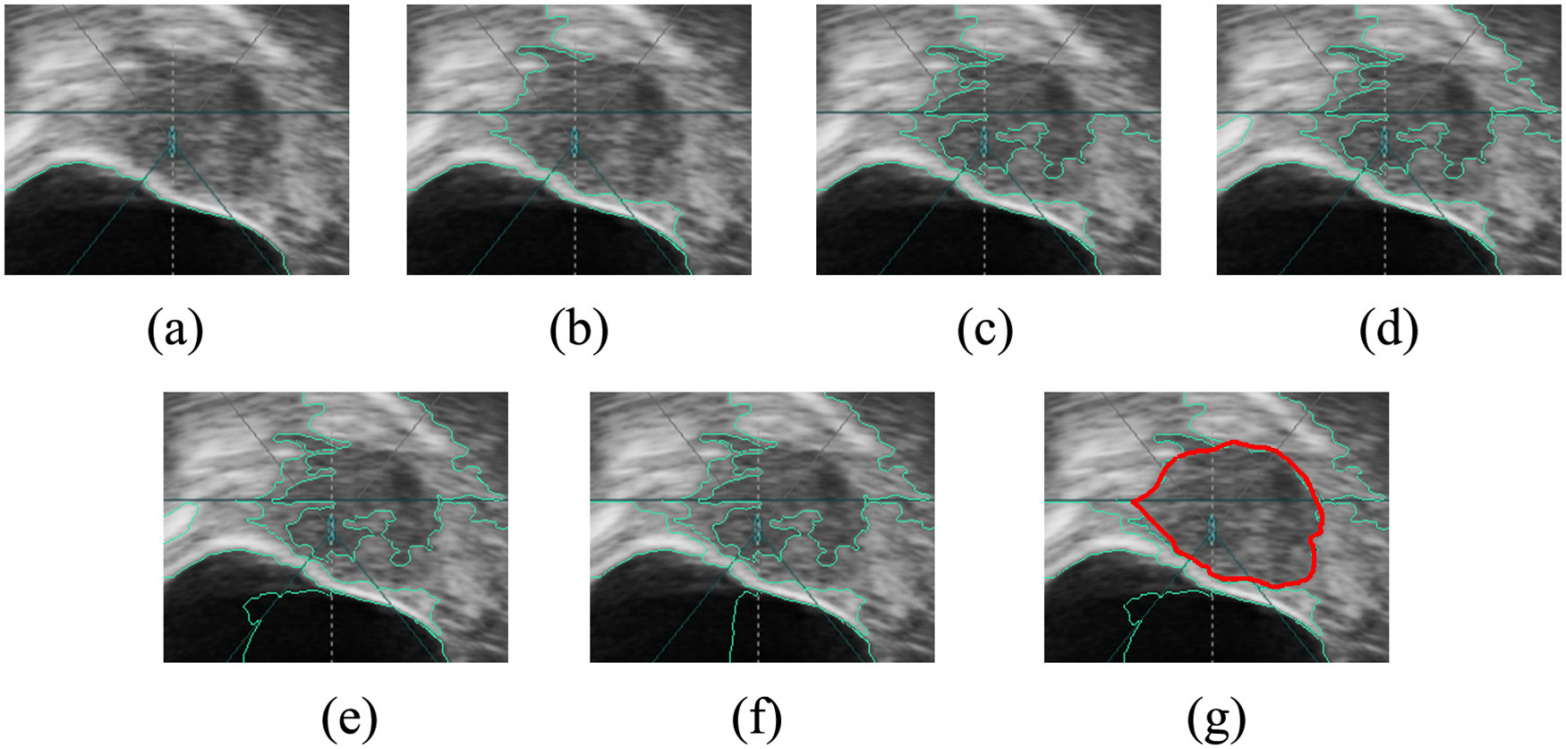
Results with different *F* values. (a) *F* = 2. (b) *F* = 3. (c) *F* = 4. (d) *F* = 5. (e) *F* = 6. (f) *F* = 7. (g) *F* = 8. When *F* = 8, the tumor is segmented. The tumor boundary is depicted with a red color.

A different number of clusters *F* leads to different results, which can be illustrated in Fig 8, for example. It is not easy to determine an appropriate number of clusters. As noted before, the uterine fibroid image always has complex structures because some other organs appear in the image as well. Thus, a simple fixed number of clusters cannot always lead to the correct segmentation of the tumor. It is tedious to adjust the appropriate number of clusters for each image manually. Thus, we developed an adaptive approach to choosing the number of clusters *F* automatically.

At the beginning of the approach, the *F* is set to 2. Next, the superpixels are clustered by Ncut. After clustering, it is tested whether the tumor region can be extracted from the merged regions. If the tumor region can be extracted, the tumor region is output, and the approach stops; otherwise, the *F* value is changed sequentially by *F* = *F*+1, and the process of clustering and testing is repeated. Three criteria are used for choosing the tumor region from the merged regions. First, the candidates should not be regions containing image boundaries. The tumor region should be complete in the ROI image. The regions containing image boundaries are regarded as incomplete because they are truncated by image boundaries; thus, those regions cannot be candidates of the tumor region. Second, after superpixel merging, the image background is always separated into several regions with very irregular shapes compared to the tumor region. Thus, tumor candidates can be chosen from irregular background regions by an ellipse-fitting algorithm. The fitting ellipse of a region is obtained from its minimum bounding rectangle [40]. The major and minor axes of the fitting ellipse, which has the same centroid with the bounding rectangle, are the width and height of the bounding rectangle, respectively. If one region is the tumor region, it should satisfy the condition that the ratio of its area to the fitting ellipse area falls into a pre-defined range. Third, from the tumor candidates, the prior tumor size is used to select the tumor region finally. In HIFU therapy, a patient always obtains many pre-operational examinations; thus, the tumor size can be known before ablation. The tumor region is the one that has the nearest area to the tumor in candidate regions that have the ratios of the area to the tumor falling into a pre-defined range. If there is no region falling into the pre-defined range, it indicates that the clustering process should be repeated with the next value of *F*. The adaptive approach is simple but performs well in practice. This adaptive approach for the automatic choice of *F* can also choose the tumor region directly to obtain the final results.

## Results

### Evaluation metrics

The proposed method is implemented by using Matlab on a PC (Intel Pentium Dual-Core CPU E5700, 2G RAM) without acceleration techniques such as parallel computing and GPU acceleration. The parameter settings are listed in Table 1. The proposed method is validated on a data set of 42 real ultrasound images from HIFU therapy described in the **Materials** subsection. The average running time is 9.54 s. Radiologists delineate the tumor boundary, which is regarded as the standard. We compared the computer segmentation results with standards delineated manually. Two metrics are used to evaluate segmentation effects, SI (similarity) and HD (Hausdorff distance).

**Table 1:** Parameter settings of the proposed method in validation.

parameter	setting
*S* ^*f*^ in bilateral filtering	13
*σ*_*d*_ in bilateral filtering	6
*σ*_*r*_ in bilateral filtering	30
*cmp* in SLIC	5
superpixel area in SLIC	1000
*r* in superpixel feature extraction	2
*K* in superpixel feature extraction	20
*σ* in the construction of *W*	0.1
pre-defined area range in the adaptive choice of the number of clusters	0.7~1.3 times of the tumor area
area ratio range in the adaptive choice of the number of clusters	0.5~1.5

The SI metric is based on overlap. Let *A*_*S*_ refer to the manually segmented region area and *A*_*A*_ refer to the automatically segmented area. SI can then be defined as follows:

$$SI=\frac{A_{S}\cap A_{A}}{A_{S}\cup A_{A}}\;.$$ (18)

A higher SI indicates much more similarity between automatic segmentation and standard segmentation. When *SI* = 1, the automatic segmentation coincides with standard segmentation. HD is another common metric based on contour distance. First, the distance between a point *u* and a closed contour *C*_*V*_ is defined as:

$$d(u,C_{V})=\underset{v\in C_{V}}{\min}\left\|u-v\right\|\;,$$ (19)

where ||*u*-*v*|| indicates the 2-D Euclidean distance between point *u* and point *v*. Next, HD between automatically segmented contour *C*_*A*_ and standard contour *C*_*S*_ is defined as

$$HD(C_{A},C_{S})=\max\left\{\max_{u\in C_{S}}d(u,C_{A}),\;\max_{v\in C_{A}}d(v,C_{S})\right\}\;.$$ (20)

HD indicates the worst contour difference between the contours of automatic segmentation and standard segmentation. Lower HD indicates more precise segmentation. However, HD is an absolute value that is not suitable to compare between different segmentations. To exclude the influence of the tumor size, the normalized form of HD [41] is used:

$$NormHD=\frac{HD}{\left|C_{S}\right|}\;,$$ (21)

where |*C*_*S*_| is the number of pixels on standard contour *C*_*S*_.

### Statistical analyses

**Fig 9:**
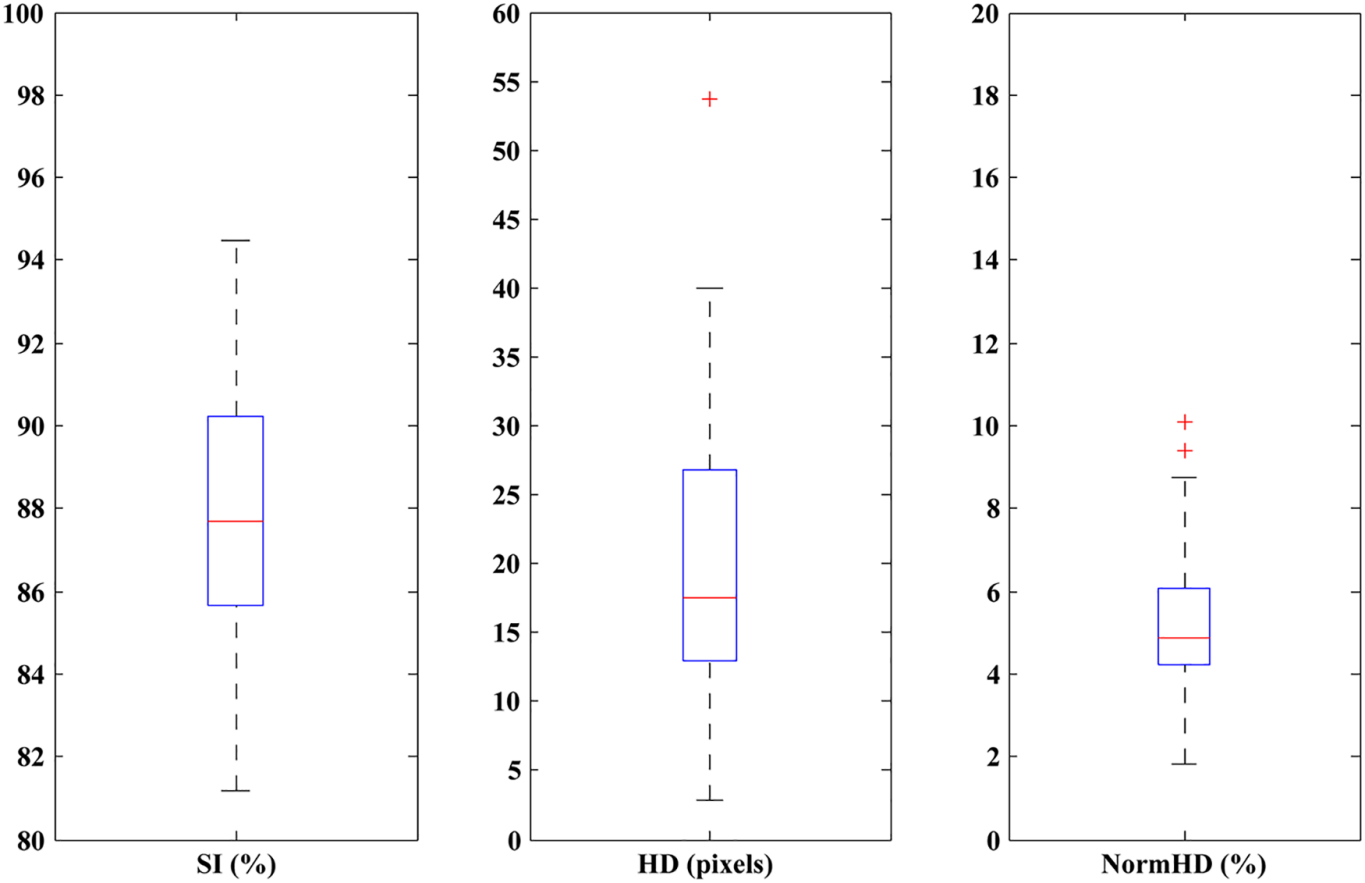
Boxplots of statistical results. Left: SI. Middle: HD. Right: NormHD.

Fig 9 shows boxplots of statistical analyses. All SI values are above 80%. The minimum SI is 81.19%, and the maximum is 94.50%. HD values are spread wider because of different tumor sizes. The minimum HD is 2.83 pixels, and the maximum HD is 53.76 pixels. NormHD excludes the influence of tumor size. NormHD values are distributed closely. Excluding the two outliers, most values of normHD distribute at approximately 5%. The minimum NormHD is 1.84%, and the maximum NormHD is 10.11%. For SI and NormHD, their lengths of boxplots are short, indicating that their values are distributed closely. The means and standard deviations are shown in Table 2. The average value of SI is 87.58%, and the NormHD is 5.18%. A high mean of SI and low means of HD and NormHD demonstrate high accuracy of segmentation. Standard deviations are low, indicating that the proposed method is robust for each test case.

**Table 2:** Means and standard deviations for SI, HD and NormHD.

	SI	HD	NormHD
**Mean**	87.58%	20.20 pixels	5.18%
**Standard deviation**	2.90%	10.50 pixels	1.68%

### Individual case study

#### Heterogeneous appearance

**Fig 10:**
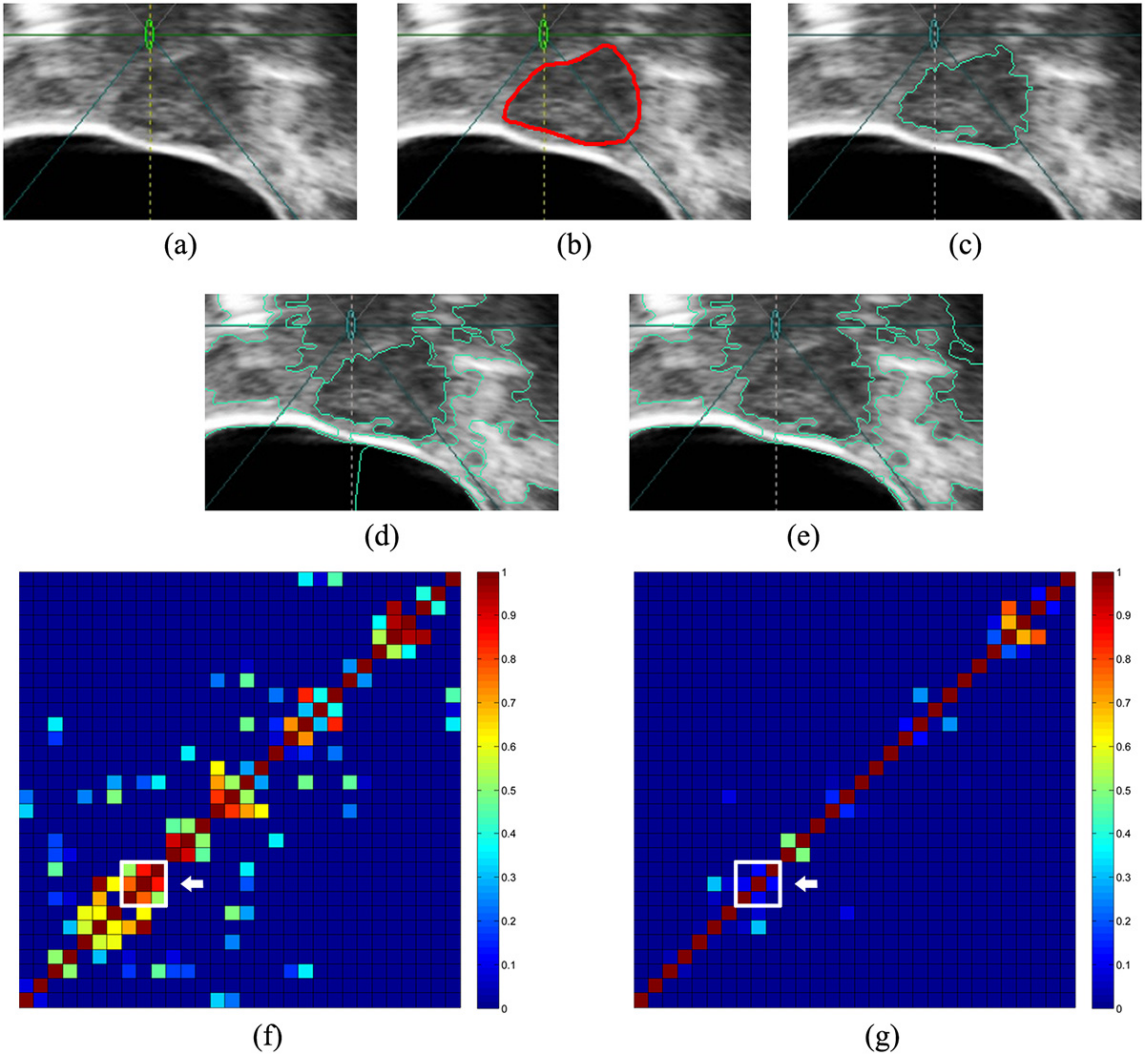
An example of a heterogeneous tumor. (a) The original image. (b) Standard boundary. (c) Boundary generated by the proposed method. (d) The merged result of the construction of *W* using the Quadratic-Chi histogram distance. (e) The merged result of the construction of *W* using the *χ* ^2^ distance. (f) Visualization of *W* using the Quadratic-Chi histogram distance. (g) Visualization of *W* using the *χ* ^2^ distance. The white arrow points to superpixels inside the tumor.

It is common for uterine fibroids to have a heterogeneous appearance, an example of which is shown in Fig 10. The gray values in the tumor region are not uniform. The proposed method segments the tumor correctly because the tumor superpixels have similar texture features that are different from other tissues. However, the successful clustering of tumor superpixels also occurs because the Quadratic-Chi histogram distance is employed. The comparison of constructing *W* using different histogram distances is shown in Fig 10. When *F* = 13, the tumor can be segmented using the Quadratic-Chi histogram distance, whereas it cannot be segmented using the *χ* ^2^ distance. Fig 10(d) and 10(e) show their merged results, respectively. Their corresponding *W* similarity matrices are also visualized using heatmaps, which are shown in Fig 10(f) and 10(g). In the heatmap, each row or column represents a superpixel. The square at the crossing point of a row and a column represents the similarity between corresponding superpixels by different colors. It can be indicated that the Quadratic-Chi histogram distance can represent similarity between superpixels more obviously than the *χ* ^2^ distance because colors of squares in Fig 10(f) spread wider than those in Fig 10(g). The squares that are indicated by white arrows represent superpixels inside the tumor. The use of the Quadratic-Chi histogram distance makes the connection of superpixels inside the tumor more obvious, a phenomenon that benefits clustering.

#### Weak boundaries

**Fig 11:**
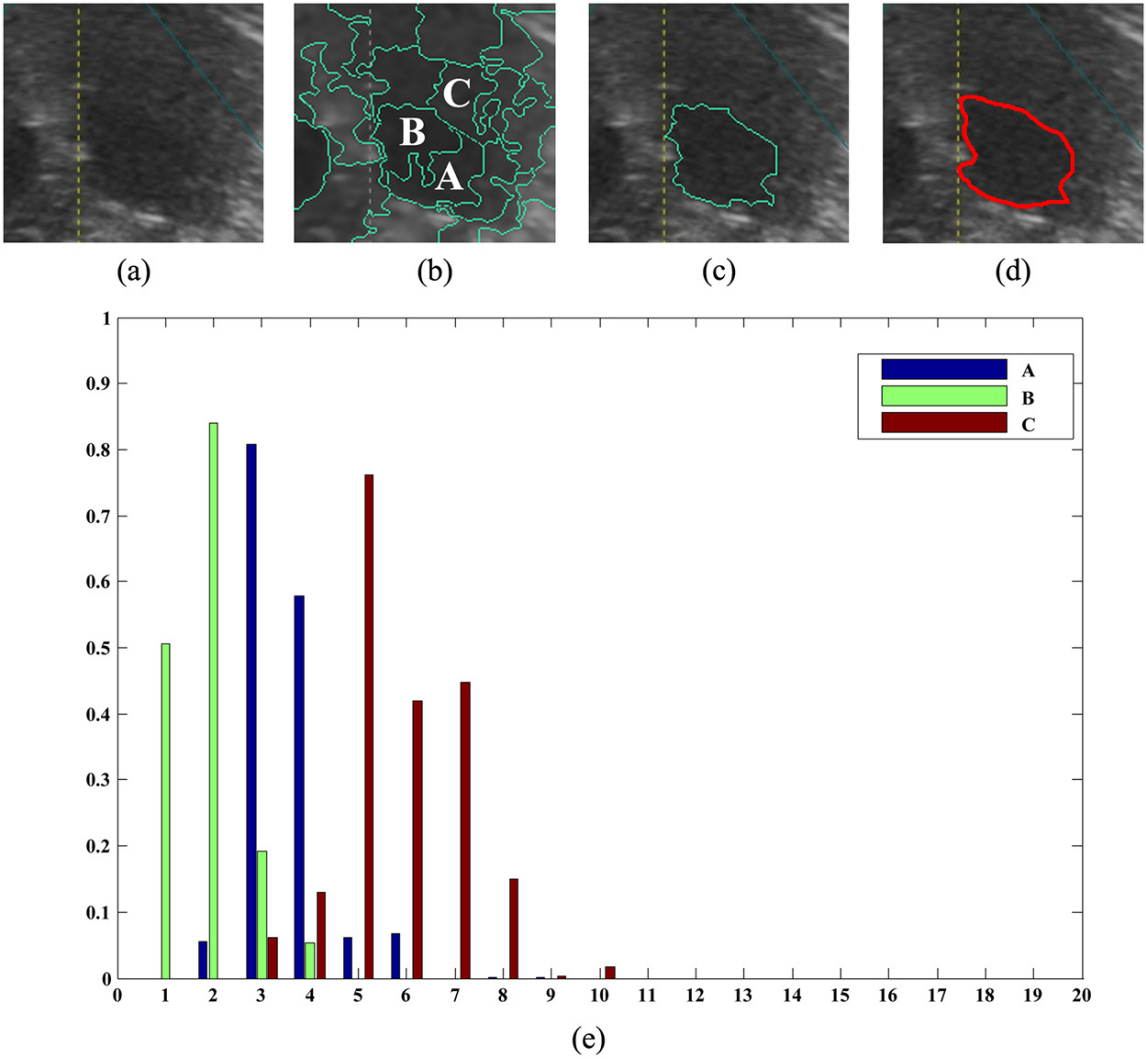
An example of a tumor with weak boundaries. (a) The original image. (b) Superpixels. Superpixels A and B are inside the tumor, while superpixel C is outside. (c) The boundary generated by the proposed method. (d) Standard boundary. (e) Histograms for three superpixels A, B, and C.

Tumors with weak boundaries are another usual case because of their acoustic characteristics. Fig 11 shows an example. The tumor boundary is too weak to be recognized. The proposed method also segments it correctly when *F* = 12. The tumor can be discriminated by texture features, although it is hard to be recognized by gray. Three texture histograms of superpixels are provided. Superpixels A and B are inside the tumor, while superpixel C is outside. They are all adjacent to each other. The histograms indicate that these superpixels have different texture features, although they look alike, because their texture histograms are not the same. Additionally, it can also be observed that the texture features of superpixels A and B are more similar than the texture features of superpixels A and C or B and C. The calculation shows that the similarity is approximately 0.82 between A and B, 0.62 between A and C and 0.42 between B and C. It can be concluded that superpixels inside the tumor have more similar texture features than superpixels outside the tumor from their histograms. Therefore, the tumor can be segmented by merging superpixels A and B according to their similarity.

## Discussion

### Experiment on synthetic data for further performance evaluation

**Fig 12:**
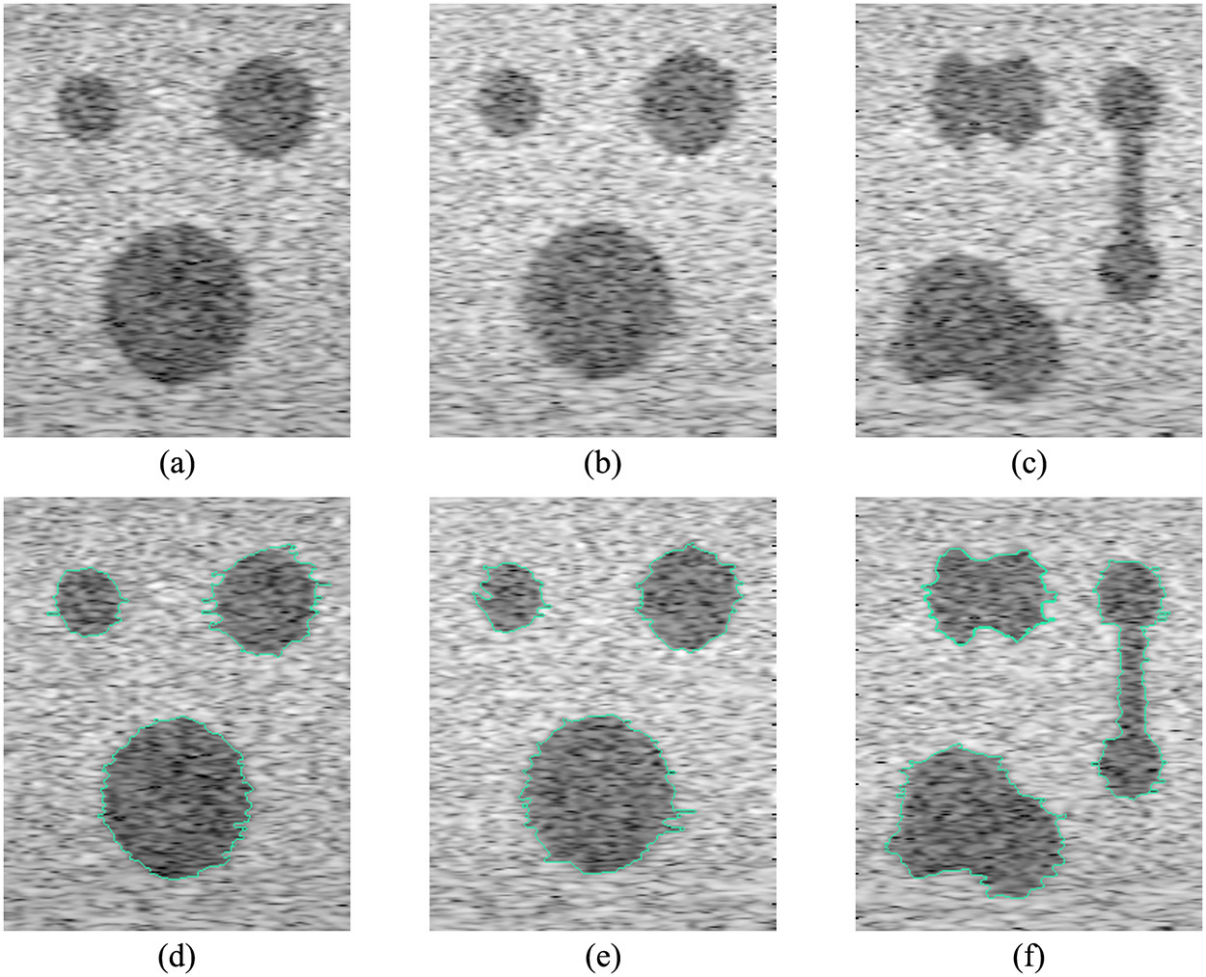
Results for synthetic images. (a) Amplitudes of scatters inside tumors have the same distribution. (b) Amplitudes of scatters inside tumors have different distributions. (c) Tumors whose amplitudes of scatters have different distributions have complex shapes. (d) Segmentation of (a). (e) Segmentation of (b). (f) Segmentation of (c).

Segmentation results demonstrate good performance of the proposed method in uterine fibroid images. To further test the performance of the proposed method in different probability distribution functions (PDFs), shapes and multi-object segmentations, we also used the method to segment synthetic images comprising subregions with differently designed PDFs, shapes and sizes that may not be included in the real image dataset. Field II [42–43] is an effective program to simulate the entire process of ultrasound imaging. We used Field II to simulate a linear transducer with a center frequency of 3.5 MHz. The segmentation results of synthetic images are presented in Fig 12. For three synthetic images, three tumors appear in each image. Each synthetic image has different simulating parameters. For the left image, the amplitude of scatters in the three tumors is set to be subject to the same normal distribution. For the middle image, each tumor has a different distribution for the amplitude of scatters. They are set to a fixed value, a normal distribution and a uniform distribution, respectively. Different amplitude distributions of scatters lead to different gray level distributions [44]. For the right image, the amplitude of scatters is set to be the same as with the middle image, but the tumors have more complex shapes. The proposed method can segment tumors correctly for all three images by setting *F* = 4. The average value of SI for synthetic images is 90.50%. The segmentation result also shows that the proposed method can handle complex situations in ultrasound image segmentation successfully.

### The best choice of *F*

**Fig 13:**
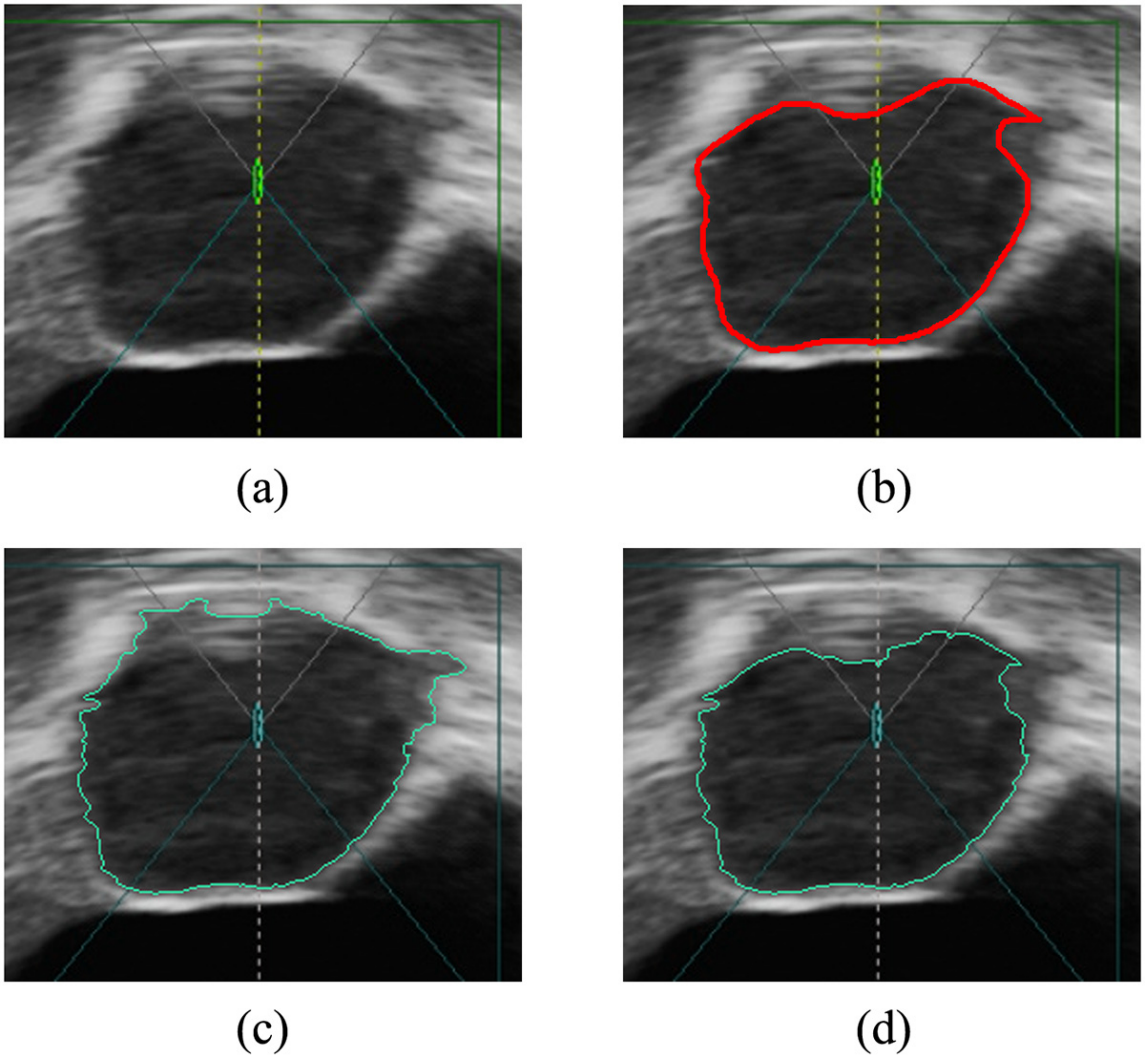
An example of tumor segmentation with different *F* values. (a) The original image. (b) Standard boundary. (c) Segmentation result when *F* = 4. (d) Segmentation result when *F* = 9.

This paper presents a novel method for uterine fibroid segmentation in HIFU therapy based on splitting and merging. Because of the complexity of uterine fibroid ultrasound images, the two-way partition by Ncut cannot always lead to correct segmentation. Thus, multi-way partition is adopted, and the merged cluster number, namely *F*, should be selected properly to obtain correct segmentation. An adaptive approach is designed to choose *F* without manual intervention. Despite its simplicity, it is demonstrated that the proposed approach performs well. Nevertheless, it should be noted that the adaptive approach cannot ensure the best *F* selection. Fig 13 shows an example. Although the tumor can be segmented correctly when *F* = 4, segmentation of the tumor with the best SI is achieved when *F* = 9. The method to obtain the best *F* is to search within all possible values. If the image has been split into *M* superpixels, the possible value of *F* is from 2 to *M*-1. It is time-consuming to search all possible values. It is observed from the experiment that the results obtained by our adaptive scheme are satisfying as well, although those results may not be the best. For example, for the tumor in Fig 13, the adaptive approach stops iteration at *F* = 4 and outputs the tumor, while the best segmentation is achieved at *F* = 9. The SI metric of the tumor region segmented with *F* = 4 is 84.29%, which is sufficiently high, although the best segmentation has a higher SI of 95.14%. It should also be noted that cases like those in Fig 13 are few in our experiment. For most images, the SI metrics of tumor regions segmented by our adaptive method are very close to the best segmentation, which is achieved due to the use of prior areas as the criterion for tumor region selection.

### Running time

The validation of both real data and synthetic data demonstrates the segmentation accuracy of the proposed method. The average running time is 9.54 s. Compared with the method using Ncut to cluster pixels [19], the proposed method is efficient because the introduction of superpixels reduces the computation complexity of clustering. However, considering that the method is applied in HIFU therapy, which requires a real-time process, it is necessary to accelerate the proposed method. The main time-consuming procedure of the proposed method is the superpixel feature extraction, which can take up over 70% of the total running time. In superpixel feature extraction, the K-Means algorithm consumes the most computing time. In K-Means, it is independent for each pixel to be assigned to a cluster; therefore, GPU acceleration, which uses multi cores of GPU to compute simultaneously, is applicable in such situations. Moreover, for SLIC, as a variant of K-Means, it is also suitable to use GPU acceleration. Our future work will be to transplant the proposed method from Matlab to CUDA (a GPU acceleration platform) with optimized codes to satisfy the real-time requirement of HIFU therapy.

### The influence of ROI

**Fig 14:**
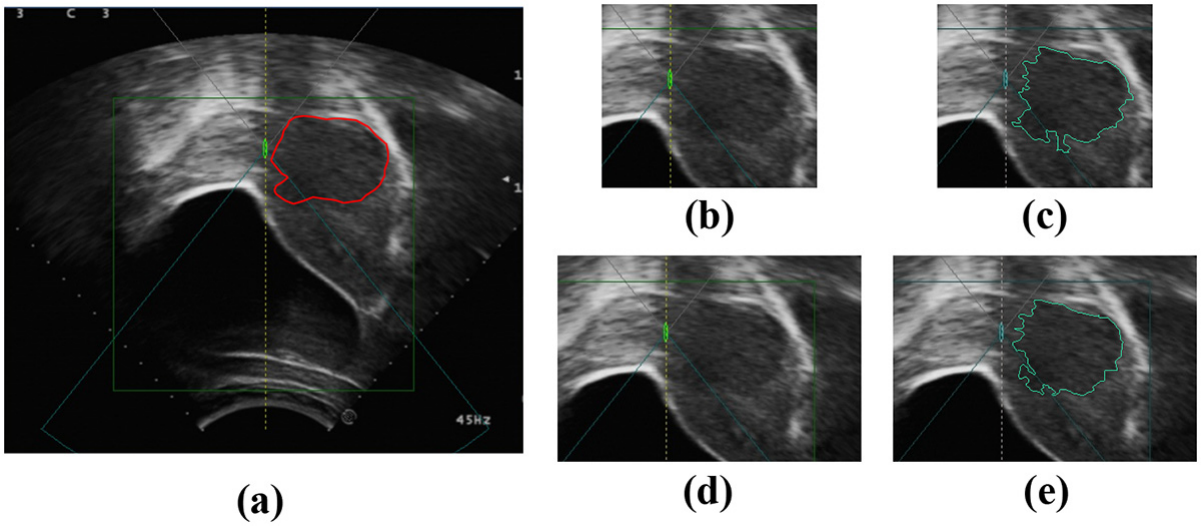
An example of tumor segmentation with a weak boundary in different ROIs. (a) The original image. (b) The first ROI image. (c) Segmentation result of (b). (d) The second ROI image. (e) Segmentation result of (d).

**Fig 15:**
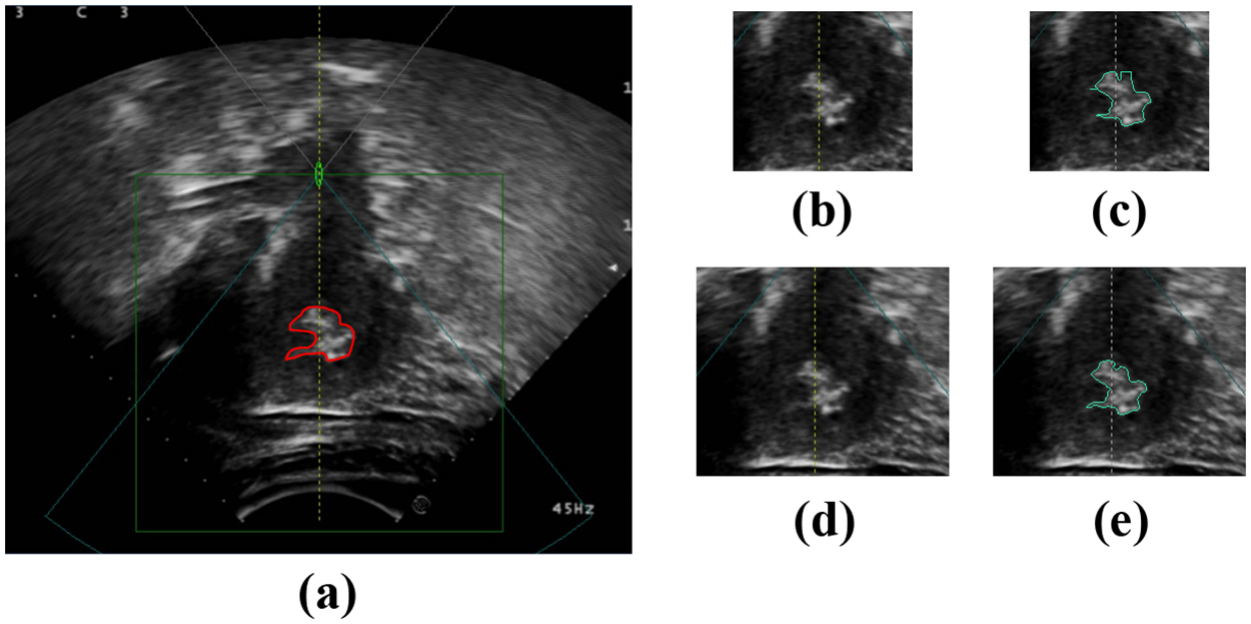
An example of tumor segmentation with hyper-echoic appearance in different ROIs. (a) The original image. (b) The first ROI image. (c) Segmentation result of (b). (d) The second ROI image. (e) Segmentation result of (d).

**Fig 16:**
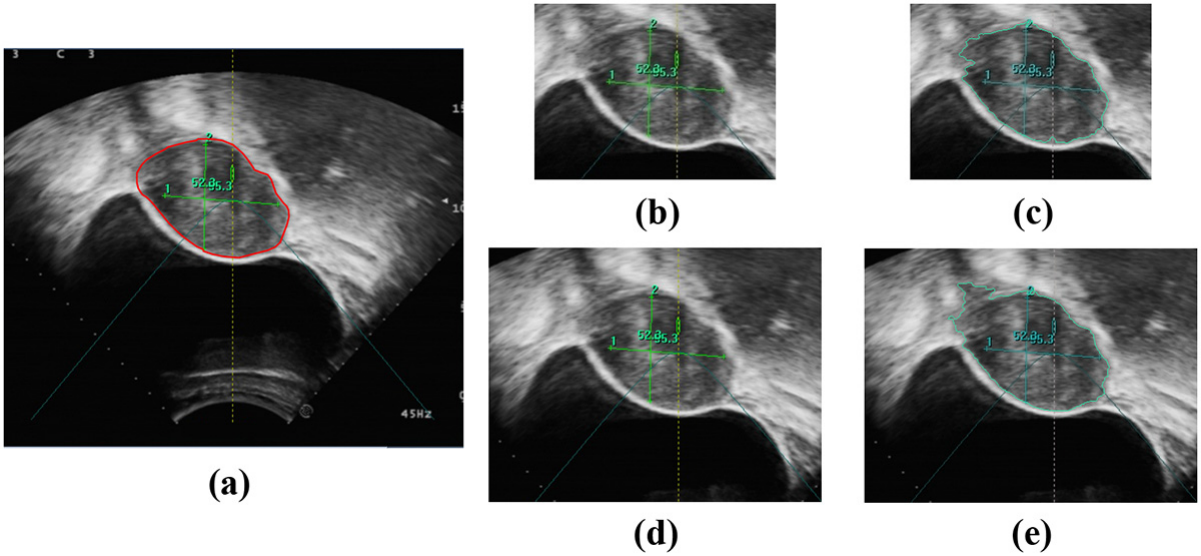
An example of tumor segmentation with great heterogeneous structure in different ROIs. (a) The original image. (b) The first ROI image. (c) Segmentation result of (b). (d) The second ROI image. (e) Segmentation result of (d).

Like many other region-based segment methods [18–19], the proposed method was tested in an ROI pre-defined on the image by a radiologist to avoid processing the image margin areas apparently unrelated with the tumor to save computing time. Of cause, the selection of ROI definitely has impact on the performance of the methods. To investigate the influence of ROI selection, another group of experiments is performed based on the new ROI drawn by another radiologist who is blinded to the former ROI selections. The average size of the new ROI is 291×337, which leads to a 1.7× increase of the ROI average area versus the former. The average running time for all images increases to 24.89 s. The results show that tumors can be segmented correctly with an average SI metric of 82.90%. Figs 14 – 16 provide examples of segmented tumors. Fig 14 shows a case of a tumor with a weak boundary. It is observed that the tumor can be segmented correctly with different ROI selections, although the segmented tumor boundaries show a slight difference. The reason for the slight change in tumor boundary is that the change in ROI causes a change in superpixels, which are generated based on the gray level and position. Although superpixels change slightly with different ROIs, they can capture the tumor boundary as well. Thus, superpixels belonging to the tumor will be merged together because they have similar texture features. Fig 15 shows the case of tumors with a hyper-echoic appearance, where the tumor can also be segmented into different ROI settings. Fig 16 shows the case with a great heterogeneous structure, in which the proposed method can perform correct segmentations in different ROIs as well.

In general, the experimental results demonstrate that the proposed method can work properly no matter what size of ROI is selected. Thus, it can be inferred that the proposed method is applicable to the original image from HIFU guidance. However, the effective area in the original image is a sector as shown in Fig 3, and it is best to define an ROI on the image to exclude the margin area and save computing time. Because the proposed method is not sensitive to the ROI selection, the ROI can be defined initially to be sufficiently large to suit for all images in the HIFU therapy process.

### Comparison with other methods

In this paper, a tumor segmentation method based on a split-and-merge algorithm is proposed for HIFU guidance images, which have lower image qualities than conventional clinical ultrasound images. However, statistical analyses have shown that the segmentation accuracy of the proposed method is at the same level with the methods already developed [11, 19] for clinical ultrasound images using the SI measure. By considering the application purpose of the proposed method, which is for automatic guidance in HIFU therapy where less manual intervention is better, a split-and-merge-based idea is proposed instead of contour evolution-based methods [11–14] to avoid manual initialization of the contour. Due to the inherent complexity of uterine fibroid ultrasound images, it is obvious that a fixed cluster number cannot be suitable for all images. Compared with the existing methods based on clustering [19, 20, 37], in which the cluster numbers are fixed or determined by implicit prior information, an adaptive method for selecting suitable cluster numbers without manual intervention is designed to ensure that the performance of the proposed method is adequate. Moreover, the algorithm of the adaptive choice of cluster numbers can choose the tumor region from merged regions without manual interaction whereas some other clustering based methods need [17, 37].

Nevertheless, unlike the methods based on contour evolution [11–14], in which the internal energy can maintain contours smooth and continuous, the segmented tumor regions in the proposed method tend to have some indentations or protuberances on the boundaries in the noisy ultrasound images used for HIFU guidance as Figs 8 and 13 show. This could potentially be improved by imposing shape constraints on the tumor region that will be the subject of the authors’ future research.

## Conclusion

A split-and-merge-based uterine fibroid segmentation method in HIFU therapy is presented in the present study. As a noninvasive treatment, HIFU has been applied in uterine fibroid treatment widely and successfully. The computerized segmentation of uterine fibroids with less manual intervention in HIFU therapy is meaningful to realize automatic localization of the target region to be ablated, which can greatly improve therapy efficiency. The proposed method splits the image into many superpixels first, thus reducing the computational complexity of the subsequent merging process. Next, each superpixel texture feature is extracted by the construction of a texture histogram. Superpixels are merged by measuring their similarities based on their Quadratic-Chi texture histogram distances and space adjacency. Multi-way Ncut is then employed in the merging process. An adaptive scheme for selecting the number of clusters is integrated with Ncut to avoid manual intervention. Compared with ultrasound image segmentation based on snakes or level sets, the proposed method does not require an initial contour, which is always obtained manually. The proposed method is validated on both real data and synthetic data. Based on an experiment with 42 real ultrasound images acquired from HIFU therapy, statistical results show that the average value of SI is 87.58%, and the normHD is 5.18%, indicating a high segmentation accuracy. It can be concluded by theoretic analysis and experimental results that the proposed method has the potential to be used in the pre-ablation imaging and planning for HIFU therapy.
